# Leiomyosarcoma of the male breast: Case report

**DOI:** 10.1016/j.amsu.2021.102495

**Published:** 2021-06-09

**Authors:** Telmoudi Ely Cheikh, Kiram Hamza, Benaguida Hicham, El Miski Fatiha, El Omri Hajar, Benhessou Mustapha, Ennachit Mohamed, Elkarroumi Mohamed

**Affiliations:** Department of Gynaecology and Obstetrics, Ibn Rochd University Hospital, Faculty of Medicine and Pharmacy, Hassan II University, Casablanca, Morocco

**Keywords:** Leiomyosarcoma, Breast, Male, Case report, Radiotherapy

## Abstract

**Introduction:**

Primary sarcoma in man is very rare and knowledge about this tumor is limited with very few cases published in the literature.

**Case report:**

A 65-year-old man operated on 20 years ago for a left breast tumor with a skin graft at the tumor site (no documentation or pathology report). He consulted for a mass in the left breast, bleeding on contact, associated with nodules in the right breast that looked suspicious. On breast ultrasound, a tissue formation in the lower medial quadrant of the left breast with irregular contours, measuring 42 × 53mm with the presence of several suspicious tissue formations in the right breast, the largest measuring 2 × 2cm.

Surgical removal of the left mass with a right mastectomy with pathology report of breast leiomyosarcoma with healthy surgical borders followed by radiotherapy for local control.

**Discussion:**

Leiomyosarcoma is an extremely rare tumor in the breast and usually originates from the blood vessels, myoepithelium, or nipple musculature, as in our case. Breast sarcomas represent less than 1% of all malignant neoplasms of the breast. Their incidence in women is much higher than in men.

**Conclusion:**

Primary breast sarcomas are rare tumors that originate from the mesenchymal tissue of the breast and represent less than 1% of all malignant neoplasms of the breast. Their diagnosis is confirmed by biopsy with immunohistochemical and only surgery can guarantee cure. Radiotherapy is recommended for local control after surgery.

## Highlights

• Primary sarcomas in man are very rare and knowledge about this tumor is limited with very few cases published in the literature.
• The diagnosis is histological, a histopathological examination followed by an immuno-histochemical analysis is very important for a precise diagnosis.
• Surgery is the only guarantee of recovery. radical mastectomy with surgical margins of at least 3cm is the standard procedure.
• Axillary lymph node dissection is not recommended.
• Radiotherapy is recommended for local control after surgical excisioN.

## 1 Introduction

Primary breast sarcomas are rare tumors originating from the mesenchymal tissue of the breast and usually occur in postmenopausal women [1]. It is a heterogeneous group that accounts for less than 1% of all primary breast tumors [2,3]. It is even rarer in men; fewer than 10 cases have been reported in men in the literature up to 2018 [4].

We present a new case of male breast leiomyosarcoma with a review of the literature and discuss the management and treatment of these unusual neoplasms. This work has been reported with respect to the SCARE 2020 criteria [5].

## 2 Case report

65 year old patient operated twenty years ago for a left breast tumor with a skin graft at the tumor site (no document or pathology report). He consulted for a mass in the left breast, bleeding on contact, which appeared three months before his consultation and had rapidly increased in volume, located opposite the initial tumor site.

The clinical examination revealed a round, pedunculated, necrotic-looking mass, bleeding on contact with the left breast, measuring 7cm in length.

**Fig. 1:**
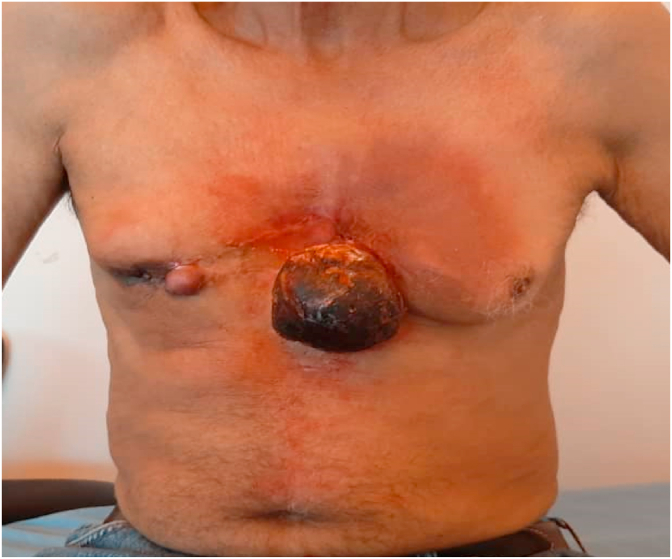
Round, pedicled mass of necrotic appearance on the left breast measuring 7 cm in long axis, inflammatory. The right breast is the site of a nodule of 3 × 2 cm associated with several small nodules in the periareolar area reaching the PAM with retraction of the nipple.

In the right breast, a 3 × 2 cm mass was present in the internal quadrants, associated with several small nodules in the internal periareolar area reaching the areola-mammary plate with retraction of the nipple. The lymph nodes were free (Fig. 1).

**Fig. 2:**
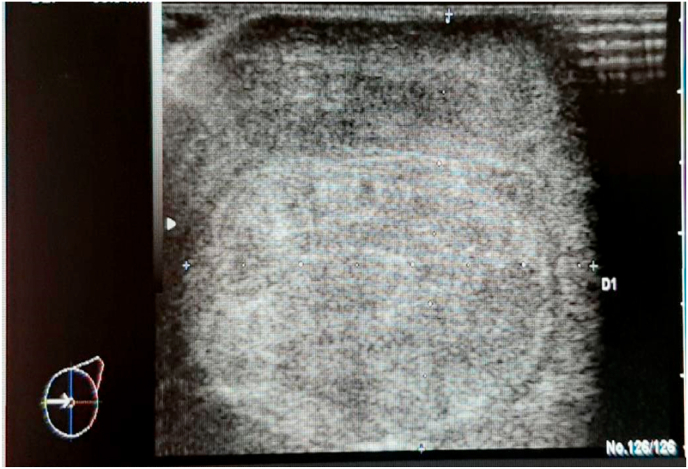
Voluminous tissue formation, with irregular contours, discreetly hypoechoic, heterogeneous echo-structure measuring 42 × 53mm, highly suspicious.

A breast ultrasound scan showed a palpable mass in the lower-internal quadrant of the left breast in the form of a large tissue formation, with irregular contours, with a discreetly hypoechoic, heterogeneous echo-structure measuring 42 × 53mm, highly suspicious with the presence of several hypoechoic, heterogeneous tissue formations, the largest of which measured 2 × 2cm. (Fig. 2).

An incisional biopsy of the mass was performed with a morphological aspect and immuno-histochemical profile in the pathology report evoking first a breast leiomyosarcoma.

Thoracic-abdominal-pelvic CT and bone scintigraphy did not show any secondary localization.

We performed a large local excision of the left mass taking away the old scar associated with a right mastectomy.

**Fig. 3:**
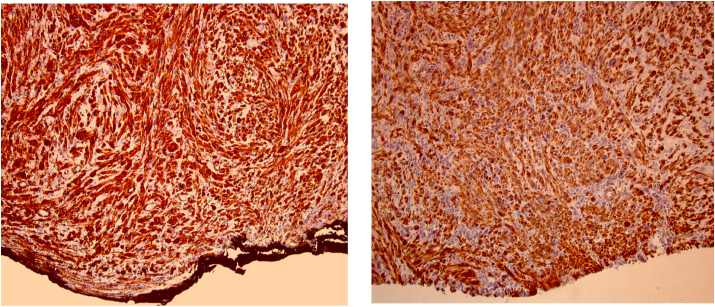
**Left breast mass: breast leiomyosarcoma** a) **IHC (AMLx100):** The cytoplasm of tumor cells expresses diffusely and intensely AML. b) **IHC (H-caldesmone x100):** The cytoplasm of the tumor cells expresses diffusely and intensely H-caldesmone.

The left ulcero-budding mass measured 8.7 × 8.5 × 7.5 cm. On section, it was soft, with a fasciculated appearance and hemorrhagic recurrences. It remained flush with the posterior border and the rest of the borders were free of tumor. Microscopic examination showed a fusocellular tumor proliferation arranged in tangled bundles with irregular elongated hyperchromatic nuclei, mitoses were estimated at 15 mitoses/10 CFG with areas of myxoid appearance. There were no vascular emboli or nerve endings. The immunohistochemical study of the surgical specimen showed intense and diffuse expression of smooth muscle actin (SMA) and h-caldesmone, without expression of Desmin, PS100, CKAE1, AE3, CD34, CD68 in favor of a breast leomyosarcoma (Fig. 3).

**Fig. 4:**
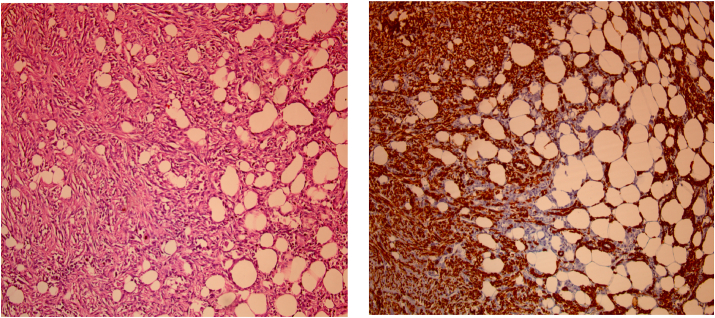
**Right mastectomy:** a) breast leiomyosarcoma (HEx100): Spindle cell tumors infiltrate adipose tissue. b) breast leiomyosarcoma **(**IHC: AML x100): Tumor cells diffusely and intensely express AML.

At the right mastectomy, a hard whitish neoplasm measuring 2.8 × 1.8 × 1.5 cm was found opposite the nipple surface. Microscopic examination showed fusocellular tumor proliferation similar to that of the left mass with immunohistochemical study showing intense and diffuse expression of smooth muscle actin (SMA) and h-caldesmone, without expression of Desmin, PS100, CKAE1, AE3, CD34, CD68 in favor of a breast leomyosarcoma. The resection margins were healthy (Fig. 4).

Radiotherapy (RT) for local control was performed at a dose of 60 Gy on the tumor bed.

**Fig. 5:**
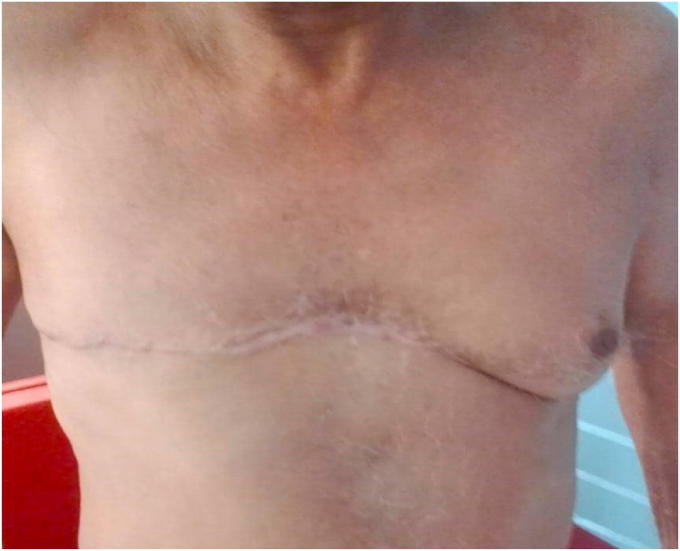
Clinical picture 11 months after radiotherapy.

The clinical evolution and the surveillance of the patient until the publication of this article, 11 months, did not show any local recurrence or general metastasis with a normal thoraco-abdomino-pelvic CT. (Fig. 5).

## 3 Discussion

The literature contains numerous examples of breast sarcomas classified largely on the basis of microscopic appearance. Despite the heterogeneity of breast sarcomas, there is a remarkable clinical similarity in this group of neoplasms. Thanks to the gradual accumulation of information through the application of electron microscopy [6].

Breast sarcomas can be separated into well-defined groups based on histogenesis and the most common subtypes are angiosarcoma and pleomorphic sarcoma, accounting for about 50% of cases.

Leiomyosarcoma is an extremely rare tumor in the breast and usually originates from the blood vessels, myoepithelium or nipple musculature, as in this case.

Breast sarcomas represent less than 1% of all malignant neoplasms of the breast. Their incidence in women is much higher than in men, until 2018 only 70 cases were reported and ten cases were men; and the first case of breast leiomyosarcoma in men was reported in the Philippines and Asia [7].

Various risk factors have been described, including a history of irradiation, chronic lymphedema, exposure to vinyl chloride, and Epstein-Barr virus (EBV) infection. At the genetic level, the 10q and 13q deleted regions harbor the two important tumor suppressor genes: RB1 and PTEN [8]. Their occurrence in males suggests the absence of endocrine factors in the genesis of these tumors [9].

Clinically, we always found a painless mass developing progressively without invasion of the deep structures of the chest wall, unlike our case, whose evolution was more or less rapid [10].

As with any breast lesion, imaging must include mammography, ultrasound and in some cases, magnetic resonance imaging of the breast. Similarly, the workup for metastatic diagnosis is the same for other malignant breast tumors. A thoraco-abdomino-pelvic CT scan is used to check for distant metastases.

The diagnosis is histological, a histopathological examination followed by immunohistochemical analysis is very important for an accurate diagnosis, It is difficult to establish a diagnosis of this rare tumor by cytology by a tru-cut biopsy [11].

Leiomyosarcoma is a solid, lobulated or large, soft tumor with areas of necrosis, hemorrhage and cystic degeneration.

Under light microscopy, this tumor presents a spindle cell morphology and is often difficult to differentiate from other tumors of the same morphology, it is characterized by alternating clusters of densely packed cells with abundant fibrillar eosinophilic cytoplasm and indistinct borders.

The centrally located nucleus is rounded and cigar-shaped. Nuclear necrosis, pleomorphism, and mitosis are additional features of the tumor [12].

The immunohistochemical profile shows a positive reaction for smooth muscle actin, vimentin, calponin, desmin and smooth muscle myosin with heavy chains and a negative reaction for S100 and CD117.

These tumors usually express smooth muscle actin, desmin and vimentin and show negative staining for S100, cytokeratins and epithelial markers [13].

Surgery is the only guarantee of cure. Radical mastectomy with surgical margins of at least 3 cm is the standard procedure, axillary lymph node dissection is not recommended since the risk of regional lymphatic extension is low and these tumors probably spread by hematogenous route to the lung, bone, liver and central nervous system.

Analysis of cases reported in the literature shows that adenopathy associated with breast leiomyosarcoma usually corresponds to hyperplasia rather than metastasis [14].

Radiotherapy is recommended for local control after surgical excision [15]. Studies have demonstrated better local control of recurrence and disease-free survival with adjuvant radiotherapy after breast-conserving resection, especially if the resection margin is marginal or insufficient. Adjuvant radiotherapy, delivered at a tumoricidal dose to the entire breast, and at least 60 Gy to the tumor bed, is recommended after positive margin resection because of the high risk of recurrence, especially if surgical revision is not possible [16]. It is also recommended for a tumor larger than 5 cm or with a high-grade sarcoma regardless of the resection margin. The good follow-up in our patient consolidates the data described in the literature.

There are less conclusive data on the use of chemotherapy. Doxorubicin, as a single agent, has a response rate of 10%–20%, however leiomyosarcomas are less responsive than other subtypes. Ifosfamide can be used as second-line therapy but has a higher toxicity profile.

Pazopanib is a new treatment option for patients with metastatic non-adipose soft tissue sarcoma after prior chemotherapy.

Chemotherapy may be recommended for high-grade tumors or those larger than 5 cm. Most trials have not shown a survival benefit from adjuvant chemotherapy in the treatment of soft tissue sarcomas. The first evidence of benefit was reported from a randomized trial for high-risk soft tissue sarcomas of the extremities and trunk wall, including leiomyosarcoma.

This trial showed an overall improved and relapse-free survival benefit in patients who received neoadjuvant therapy [17].

In general, the prognosis of patients with breast leiomyosarcoma is considered better than that of patients with other breast sarcomas [18,19].

The 5-year survival rates are 63%, 36%, and 14% for localized, regional, and distant disease, respectively. However, because there is a high risk of recurrence, long-term follow-up may be indicated [20].

## 4 Conclusion

Leiomyosarcoma is an extremely rare tumor of the breast and usually originates from the blood vessels, myoepithelium, or nipple musculature. Breast sarcoma accounts for less than 1% of all malignant neoplasms of the breast. It is even rarer in men.

Surgery is the only guarantee of cure; it consists of a radical mastectomy with surgical margins of at least 3cm, followed by radiotherapy for local control.

## Ethical Approval

I declare on my honor that the ethical approval has been exempted by my establishment.

## Author contribution

Telmoudi Ely Cheikh: Corresponding author writing the paper and operating surgeon, Kiram Hamza: writing the paper and operating surgeon, Benaguida Hicham: study concept, El Miski Fatiha: study concept, El Omri Hajar: study concept, Benhessou Mustapha: study concept, Ennachit Mohamed: study concept, Elkarroumi Mohamed: correction of the paper and operating surgeon.

## Registration of research studies

researchregistry2464

## Guarantor

DR TELMOUDI ELY CHEIKH

## Consent

Written informed consent for publication of their clinical details and/or clinical images was obtained from the patient.

## Provenance and peer review

Not commissioned, externally peer-reviewed.

## Funding

None.

## Declaration of competing interest

The authors report no declarations of interest.
